# Structural basis for proton coupled cystine transport by cystinosin

**DOI:** 10.1038/s41467-022-32589-2

**Published:** 2022-08-17

**Authors:** Mark Löbel, Sacha P. Salphati, Kamel El Omari, Armin Wagner, Stephen J. Tucker, Joanne L. Parker, Simon Newstead

**Affiliations:** 1 Department of Biochemistry, University of Oxford, Oxford, UK; 2 Clarendon Laboratory, Department of Physics, University of Oxford, Parks Road, Oxford, UK; 3 Diamond Light Source, Harwell Science and Innovation Campus, Didcot, UK; 4 Kavli Institute for Nanoscience Discovery, University of Oxford, Oxford, UK

**Keywords:** X-ray crystallography, Structural biology

## Abstract

Amino acid transporters play a key role controlling the flow of nutrients across the lysosomal membrane and regulating metabolism in the cell. Mutations in the gene encoding the transporter cystinosin result in cystinosis, an autosomal recessive metabolic disorder characterised by the accumulation of cystine crystals in the lysosome. Cystinosin is a member of the PQ-loop family of solute carrier (SLC) transporters and uses the proton gradient to drive cystine export into the cytoplasm. However, the molecular basis for cystinosin function remains elusive, hampering efforts to develop novel treatments for cystinosis and understand the mechanisms of ion driven transport in the PQ-loop family. To address these questions, we present the crystal structures of cystinosin from *Arabidopsis thaliana* in both apo and cystine bound states. Using a combination of in vitro and in vivo based assays, we establish a mechanism for cystine recognition and proton coupled transport. Mutational mapping and functional characterisation of human cystinosin further provide a framework for understanding the molecular impact of disease-causing mutations.

## Abstract (web summary)

Mutations in CTNS, the lysosomal cystine-proton symporter, cause cystinosis. Here authors report crystal structures of CTNS from Arabidopsis thaliana in complex with cystine, and establish the mode of ligand recognition and mechanism for proton-coupled cystine export from the lysosome.

## Introduction

The lysosome is the main site of macromolecule degradation in mammalian cells and functions as a major signalling centre, integrating multiple inputs to regulate cellular metabolism via mTOR^1–3^. Central to lysosomal function and cell homoeostasis is the transport of amino acids across the lysosomal membrane via solute carrier (SLC) systems^4–6^. Solute carrier proteins are secondary active transporters that control the flow of nutrients between and within cells, often linked to ion gradients that function to concentrate nutrients and regulate levels in response to cellular energy demands^7–9^. Dysregulation of SLC function in the lysosome is linked to several lysosomal storage disorders, which are inherited metabolic diseases resulting from defective lysosomal function^10^. Mutations in the gene encoding cystinosin, *CTNS*, result in cystinosis, an autosomal recessive metabolic disease characterised by abnormal accumulation of cystine, the oxidised form of cysteine, inside lysosomes^11,12^. The *CTNS* gene was identified in 1998 and to date more than 140 mutations have been linked to nephropathic cystinosis in patients around the world^11,13^. Cystinosis often leads to renal Fanconi syndrome in paediatric patients, characterised by reduced kidney function and impaired growth and bone development due to mineral depletion^14^. Less severe forms of the disease result in blindness due to cystine crystallisation in the cornea, referred to as ocular cystinosis^15^. Defective lysosomal cystine transport also contributes to acute cellular oxidative stress, mitochondrial dysfunction, trafficking defects, and proteolysis defects in the cell^13,16,17^. However, a mechanistic link between cystinosis symptoms in patients and mutations in the *CTNS* gene is not fully established at the molecular level, hampering efforts to develop new treatments^18^.

**Fig. 1:**
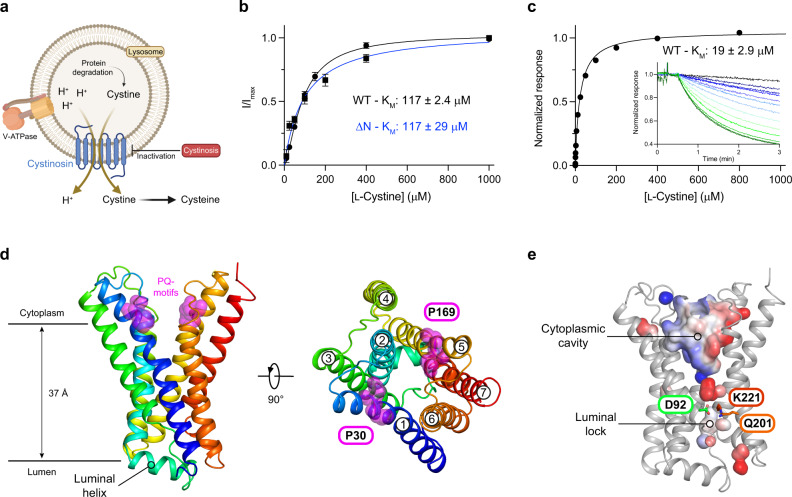
Functional characterisation and structure determination of cystinosin. **a** Cystinosin resides within the lysosomal membrane where it is responsible for the export of cystine from the lysosomal lumen. Transport is proton coupled, utilising the proton gradient across the membrane generated through the action of V-ATPase. Mutation of the CTNS gene, which encodes cystinosin, lead to cystinosis. **b** Representative K_M_ curve derived from TEVC for full length human cystinosin (WT – black) and a construct lacking N-terminal amino acids 2-115 (ΔN – blue). *n* = 16 independent experiments for every WT data point, *n* = 10 for ΔN, error bars SEM. K_M_ values were calculated from three biologically independent experiments, error SD. **c** Representative K_M_ analysis of plant cystinosin derived using a pyranine based in vitro assay. K_M_ was calculated from three independent experiments, error SD. Inset shows a typical set of raw data generated from the assay (lines are coloured according to cystine concentration dark green 800 via lighter colours to dark blue 0 μM). **d** Crystal structure of plant cystinosin with helices coloured blue to red from the N-terminus. Highlighted (magenta spheres) are the two PQ loop motifs which sit in the open cavity facing the cytoplasm in the conformation captured in the structure. **e** View of cystinosin highlighting the salt bridge network which forms the luminal lock and seals the binding site on the luminal side. Source data are provided as a Source Data file.

Cystinosin is a proton coupled lysosomal cystine transporter^19^, which functions as the primary route for the export of cystine out of the lysosome (Fig. 1a). The transporter comprises seven transmembrane helices (TMs) and uses the outwardly directed proton electrochemical gradient, generated by the V-ATPase, to drive cystine export from the lysosome in a 1:1 stoichiometry^20^. Cystinosin is also a member of the so-called PQ-loop family^21,22^, members of which function as trafficking receptors^23^, lipid flippases^24^, amino acid transporters^25^ and sugar transporters in plants and mammals^26^. Eukaryotic PQ-loop proteins contain two PQ-motifs, located on TM1 and TM5, which play important roles in facilitating the structural changes required for alternating access transport within these proteins^22,27^.

Insights into the structural changes that occur in transporters belonging to the PQ-loop family were revealed through crystal structures of the bacterial PQ-loop transporters, which were captured in both the inward (cytoplasmic) and outward (extracellular) states of their transport cycles^27–29^. However, unlike eukaryotic PQ-loop family transporters, the bacterial homologues are obligate dimers, consisting of two 3-TM domains and function as facilitators, allowing sugar molecules to equilibrate across the membrane^30^. Although a plant homologue of the bacterial semi-SWEET transporters was also crystallised^31^, revealing the architecture of the eukaryotic hepta-helical PQ-fold, we still have little insight into how PQ-loop transporters couple ligand binding and transport to secondary ion gradients. Previous work on human cystinosin, using two electrode voltage clamp (TEVC) assays, revealed the presence of several conserved aspartate residues, one of which undergoes reversible protonation during transport^20^. Cystine binding was proposed to alter the pK_a_ of this side chain, providing a mechanistic link for coupled transport. However, several key questions concerning the mechanism of cystinosin remain, including how cystine is recognised, how proton binding and release drive the full transport cycle and the rationalisation of patient mutations within the mechanism of transport.

To address these questions and understand the molecular basis for proton coupled cystine transport, we determined the structure of the plant cystinosin transporter from *Arabidopsis thaliana*. Using both Llama derived^32^ and synthetic nanobodies^33^, cystinosin was captured in both apo and ligand bound states, revealing a key role for conserved lysine side chains in cystine recognition. Combined with in vitro transport assays and TEVC comparisons on the human transporter we propose a mechanism for proton coupled cystine transport across the vacuolar and lysosomal membranes.

## Results

### Characterisation of *Arabidopsis thaliana* cystinosin

To identify homologues of cystinosin suitable for structural and biochemical analysis we screened several homologues from different organisms (Supplementary Fig. 1a). The homologue from *Arabidopsis thaliana* showed high expression levels in our yeast-based system^34^ relative to other homologues and was therefore chosen for further analysis. Cystinosin from *A. thaliana*, however, does not contain the N-terminal lysosomal luminal domain found in human and other eukaryotic cystinosin proteins (Supplementary Fig. 1b), prompting us to determine the importance of this domain for transport function. Analysing the human transporter expressed in *Xenopus laevis* oocytes and measuring currents elicited by l-cystine under two electrode voltage clamp (TEVC), we found that the N-terminal domain of the human transporter does not impact the transport kinetics, with both having similar K_M_ values for cystine (117 ± 2.4 μM for WT compared to 117 ± 28.9 μM for the Δ2-115 variant) (Fig. 1b & Supplementary Fig. 2a). We then determined the K_M_ for cystine transport using a reconstituted system and employed pyranine to monitor proton movement^35^. These results establish *A. thaliana* cystinosin as a proton coupled cystine symporter with a K_M_ for cystine of 19 ± 2.9 μM, (Fig. 1c & Supplementary Fig. 2b) suggesting a slightly higher affinity for l-cystine than the human protein. Having established the plant transporter as a suitable functional homologue of human cystinosin, we sought to determine the structure using crystallisation in the lipidic cubic phase.

### Structure of cystinosin

Single chain binders have been successfully used to obtain the structures of small, dynamic membrane proteins^36^. Using a similar strategy to our recent work on the KDEL receptor^23^, we screened both a synthetic nanobody (sybody) library and a library generated via llama immunisation to identify specific binders to cystinosin (Supplementary Fig. 3). The structure was initially determined in complex with both a cytoplasmic binding sybody and luminal binding nanobody to 2.65 Å and phases calculated using long wavelength sulfur single wavelength anomalous dispersion (S-SAD) (Table 1 & Supplementary Fig. 4a). The transporter adopts a compact V-shaped structure, consisting of seven transmembrane (TM) alpha helices and adopts an inward (cytoplasmic) open state, with the CDR3 loop of the sybody (Syb39) extending into the cytoplasmic entrance of the binding site (Fig. 1d & Supplementary Fig. 4b). On the luminal side of the transporter the llama derived nanobody (Nb4) interacts with the N-terminus of TM1 and the luminal end of TM7 (Supplementary Fig. 4a, b). Within the crystal the binders act to bridge two of the transporters together, aiding crystal packing within the lipidic cubic phase. We also identified a second binder combination, consisting of two llama nanobodies, which diffracted to a slightly higher resolution of 2.33 Å (Table 1). The structures of the transporter in both crystal forms are very similar (root mean square deviation (RMSD) of 0.776 Å over 224 C_α_ atoms), with the only difference being loss of density for the cytoplasmic end of TM4 and the connecting loop to TM5 in the 2.33 Å structure (Supplementary Fig. 4c, d).

**Table 1:** X-ray data collection and refinement statistics

		S-SAD data	Cystinosin apo (Syb39 & Nb4)	Cystinosin apo (Nb1 & Nb4)	Cystinosin cystine complex (Syb39)
Data collection	PDB	–	7ZK1	7ZKZ	7ZKW
	Space group	*P* 2_1_ 2_1_ 2	*P* 2_1_ 2_1_ 2	*C* 2	*C* 2
	Cell dimensions a, b, c (Å)	62.60, 319.49, 45.48	62.79, 319.96, 45.65	210.28, 77.46, 46.30	283.37, 64.07, 55.44
	Cell angles α, β, γ (°)	90, 90, 90	90, 90, 90	90, 93.25, 90	90, 99.95, 90
	Wavelength (Å)	2.7552	0.9999	0.9795	0.9762
	Resolution (Å) ^a^	29.24–3.20 (3.28–3.20)	79.99–2.65 (2.69–2.65)	104.76–2.33 (2.37–2.33)	70.10–3.37 (3.52–3.37)
	CC1/2 (%) ^a^	99.9 (67.1)	99.8 (35.7)	99.5 (42.7)	79.7 (10.0)
	R_pim_ (%) ^a^	5 (99.2)	11.4 (227.6)	7.2 (91.7)	49.7 (120.5)
	I/σI ^a^	13.7 (1.1)	5.9 (0.4)	7.9 (0.6)	2.0 (0.6)
	Completeness (%) ^a^	99.7 (99.5)	100 (99.9)	100 (100)	99.7 (95.3)
	Multiplicity ^a^	44.6 (38.6)	23.9 (13.3)	6.4 (6.7)	6.1 (5.9)
Refinement	Resolution (Å)		79.99–2.65	20.79–2.33	69.78–3.37
	Number of reflections		26170	31641	12426
	R_work_/R_free_		23.4/27.49	23.5/26.5	23.5/27.7
	F_o_,F_c_ correlation		0.83	0.94	0.83
	Average B, all atoms (Å^2^)		84.8	66.9	67.5
R.m.s deviations	Bond lengths (Å)		0.009	0.008	0.014
	Bong angles (°)		1.05	0.97	1.55
	Ramachandran statistics Favoured/outliers (%)		91.55/2.47	97.88/0.64	89.72/2.50
	Molprobity score		2.97	1.77	2.94

^a^Highest resolution shell shown in parentheses.

The structure has similarities to both the eukaryotic SWEET transporter, the bacterial semi-SWEET transporters and KDEL receptor^23,28,29,31^, although with some notable differences. Cystinosin aligns with the previously determined SWEET transporter from *Oryza sativa* (rice) with an RMSD of 4.9 Å over 208 C_α_ atoms, which also adopts an inward open conformation^31^. However, the inversion helix (TM4) is positioned closer to TM5 forming a more spherical molecule. Another notable difference observed in cystinosin is the presence of a luminal helix connecting TM2 and TM3. This feature is likely to be present in all cystinosin homologues given the sequence conservation in this region (Supplementary Fig. 1b) and as discussed below, may play an important role in the transport mechanism by stabilising a shortunwound region of TM3. PQ-loop transporters contain a pair of three TM bundles, TMs 1-3 and TMs 5-7, which frame a central binding site^27^. In the structure of cystinosin, a polar cavity extends 23 Å into the centre of the transporter from the cytoplasmic side of the molecule, which contains a high density of positively charged side chains (Fig. 1e). The positive charges continue down into the binding site, which is dominated by the presence of two lysine side chains Lys55 (TM2) and Lys166 (TM5) and His56 (TM2), which as discussed below play an important role in cystine recognition. The luminal side of the transporter is sealed through the packing of TMs 3, 6 and 7, which are stabilised through interactions between Asp92 (TM3), Gln201 (TM6) and Lys221 (TM7) (Fig. 1e). This interaction network is strictly conserved within cystinosin homologues and as discussed below plays an important role in the transport mechanism.

### Molecular basis for cystine recognition

**Fig. 2:**
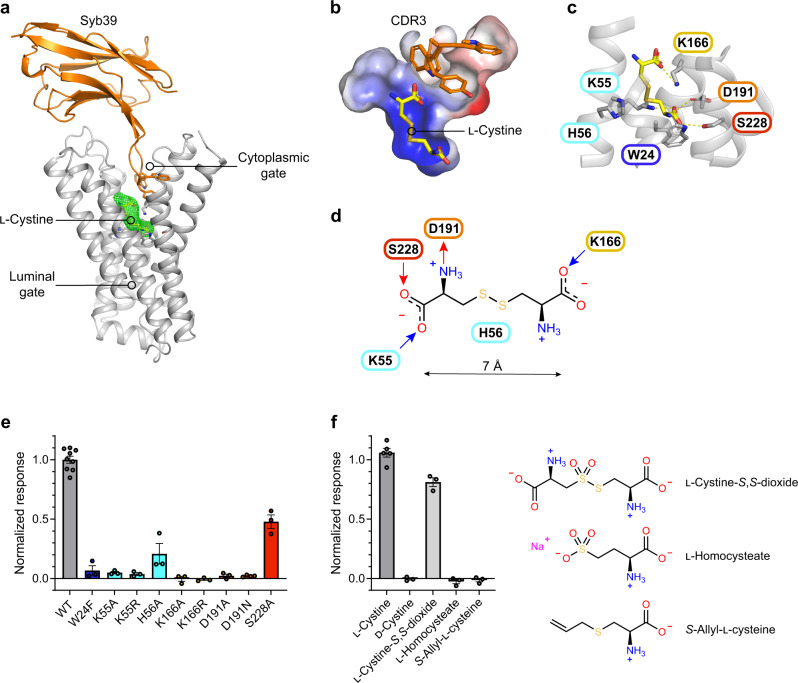
Structural basis for l-cystine recognition. **a** Crystal structure of cystinosin showing sybody 39, which aided in trapping the cystine ligand within the binding site. Cystine mFo-DFc difference electron density map (green mesh) contoured at 3σ. **b** The central binding site is positively charged complementing the negative charge on cystine. **c** Zoomed in view of the binding site showing the interaction network between cystinosin and cystine. **d** Schematic of cystine highlighting key interactions in the binding site. **e** Impact of binding site variants on cystine transport using a pyranine based in vitro assay. All variants were tested using 200 μM l-cystine. *n* = 3 independent experiments for variants (*n* = 4 for D191N) and *n* = 9 for WT, error bars SEM. **f** Substrate specificity of cystinosin analysed using a pyranine based in vitro assay system. All substrates were tested at 500 μM. *n* = 5 for l-cystine and *n* = 3 for all other substrates, error bars SEM. Source data are provided as a Source Data file.

To understand the structural basis of the recognition and transport of cystine we employed our previous binder trap methodology to stabilise the ligand bound state for structure determination^37^. Following several rounds of screening with different binder combinations we determined the structure of the ligand bound state in complex with Syb39 to 3.37 Å (Table 1). The ligand bound conformation of cystinosin is very similar to the apo state, with an RMSD of 0.48 Å over 240 C_α_ atoms. The cystine ligand was clearly resolved in the electron density map and sits at the base of the polar pocket observed in the apo state (Fig. 2a, b). Cystine adopts an extended conformation within the binding site and makes several notable interactions to conserved side chains (Fig. 2c, d). Cystine can exist in three different ionic forms depending on the pH of the environment^38^. Within physiological pH the carboxyl groups will be fully dissociated, however, the two amino groups have different pKa values of 7.48 and 9.02. Our crystallisation trials were conducted at pH 6.5, favouring the zwitterionic form of cystine, which we modelled into the structure. Indeed, we observe a salt bridge interaction between one of the amino groups of cystine and Asp191 (TM6). In addition, we observe two further salt bridge interactions between the two carboxylate groups and Lys55 (TM2) and Lys166 (TM5). A further hydrogen bond interaction is made to a conserved serine, Ser228 (TM7). The disulfide bond is sandwiched between Lys166 (TM5) and His56 (TM2), which would help to stabilise the delocalised negative charge, whereas the second amino group makes no specific interactions and points towards a hydrophobic cavity between TM2 and TM3.

To assess whether these observed interactions are important for l-cystine transport, we generated a series of variants in the plant transporter and tested their function using both a pyranine-based transport assay and a differential scanning fluorimetry (nanoDSF) binding assay. We found that alanine variants of the main interaction sites Lys55, Lys166 and Asp191, resulted in inactive protein in both assays (Fig. 2e and Supplementary Fig. 5). Furthermore, even conservative mutations of these side chains to arginine and asparagine respectively, could not recover transport activity, highlighting their importance to the mechanism. Interestingly the tryptophan, Trp24, which sits at the base of the binding site, is also important for l-cystine uptake, with even a conservative mutation to phenylalanine only exhibiting ~10% WT transport levels. In the binding assay this variant loses ~50% binding. Together these results suggest a possible cation-π interaction with the amino group that sits ~3.4 Å away in the binding site and demonstrates an essential role for Trp24 in the conformational changes required for transport. The His56Ala variant retained ~20% transport activity, indicating that this side chain is not essential for l-cystine recognition. l-Cystine also contacts Ser228 in the base of the binding site. An alanine variant of this side chain reduces both binding and transport by ~50%, indicating this side chain plays only a supporting role in transport (Fig. 2e and Supplementary Fig. 5).

A noticeable feature of the l-cystine binding pose are the interactions observed between the two carboxylate groups of the ligand and the Lys55 and Lys166 side chains. To assess the importance of these interactions we screened a library of structural analogues of l-cystine (Fig. 2f). We observed that only l-cystine-*S*,*S*-dioxide, which contains two carboxylates could be transported, at ~80% WT levels and consistent with previous assays conducted using the human transporter^20^. The uptake of l-cystine-*S*,*S*-dioxide also suggests that the position of the disulfide group is likely to be flexible to accommodate the additional two carbonyl groups which bind to one sulfur atom of the disulfide, consistent with our crystal structure. Interestingly, neither *S*-allyl-l-cysteine, which has an amino acid group replaced with an allyl group, nor l-homocysteate, which contains a sulfonate group in place of the second l-cysteine moiety, could be transported. These results show that the length of the ligand, in addition to the presence of the two carboxylate groups is important. Finally, d-cystine was also not transported, demonstrating that cystinosin is stereospecific. These results thus establish a preliminary binding model for plant cystinosin (Fig. 2d) emphasising the importance of the two salt bridges and an ideal distance of ~7 Å between the two carboxylate groups.

**Fig. 3:**
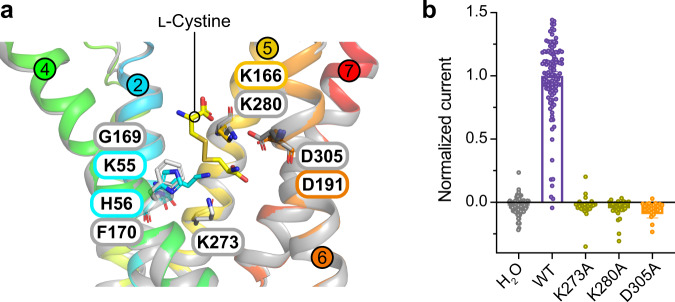
Differences in the binding site between plant and human cystinosin. **a** Structural superimposition of the plant crystal structure (coloured helices and numbers) and human AlphaFold2 model (grey) revealing the movement of key residues within the binding site. **b** TEVC data showing the effect of mutations within the binding site of human cystinosin. *n* = 109 independent experiments for H_2_O, *n* = 113 for WT, *n* = 28 for K273A and D305A, *n* = 30 for K280A, error bars SEM. Source data are provided as a Source Data file.

We next compared our structure to that predicted by AlphaFold2 for the human protein^39^. Many of the side chains identified in the binding site of plant cystinosin are strictly conserved within the human transporter, nevertheless, notable differences exist. In human cystinosin the equivalent side chain to His56 is phenylalanine (Phe170) and Lys55 is a glycine (Gly169) (Supplementary Fig. 1b). However, the AlphaFold2 model shows that in the human transporter a lysine, Lys273, extends from TM5 to sit in a similar position in the binding site (Fig. 3a). Using our oocyte transport assay, we tested the Lys273Ala variant and found this lysine is essential for function (Fig. 3b & Supplementary Fig. 6), similar to the structurally equivalent lysine in plant cystinosin. We then analysed the alanine variants of Lys280 and Asp305, which are the equivalent side chains to Lys166 and Asp191 in the plant transporter. These side chains are also essential for l-cystine transport, highlighting an evolutionary conserved mechanism of l-cystine recognition.

### Mechanism of cystine transport

**Fig. 4:**
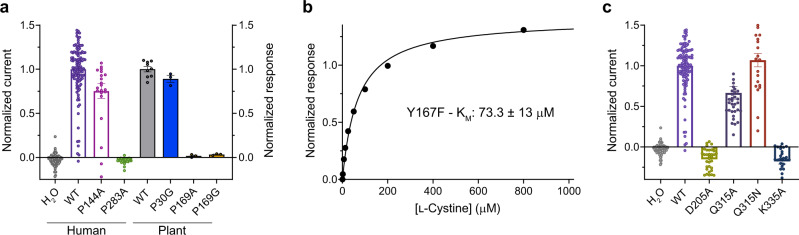
Role of PQ motifs in cystinosin. **a** Effect of mutating the PQ motifs in both human and plant (*A. thaliana*) cystinosin. The human protein was assayed using TEVC (*n* = 109 independent experiments for H_2_O, *n* = 113 for WT, *n* = 22 for P144A and *n* = 31 for P283A) whereas the plant transporter was analysed in reconstituted liposomes (*n* = 9 independent experiments for WT and *n* = 3 for variants). Error bars SEM. **b** Representative K_M_ of plant cystinosin variant Tyr167Phe. K_M_ was calculated from three independent experiments, error SD. **c** Summary data showing the effect of mutations on the proposed luminal lock of human cystinosin expressed in Xenopus oocytes. *n* = 109 independent experiments for H_2_O, *n* = 113 for WT, *n* = 35 for D205A, *n* = 34 for Q315A, *n* = 22 for Q315N, *n* = 27 for K335A, error bars SEM. Source data are provided as a Source Data file.

Secondary active transporters operate via an alternating access mechanism, whereby the transporter switches between outward and inward facing states to move ligands and ions across the membrane^40^. Within the PQ-loop family, alternating access transport is understood to occur following the symmetrical movement of helices within the two 3TM bundles^22^. Specifically, the PQ-motifs on TM1 and TM5 are required to enable the bending of these helices to open and close the intracellular gate in response to ligand and/or ion binding. In the plant SWEET transporter, the glutamine residues are absent from both PQ-motifs, with the transporter still functional in cells, however, the prolines were essential for function^31^. Therefore, to understand the role of the prolines within the transport mechanism of cystinosin, we assayed the proline to alanine variants in the human and plant transporters (Fig. 4a). In human cystinosin the proline of the first PQ-motif is dispensable, with transport levels of an alanine variant exhibiting ~75% WT levels. The equivalent alanine variant in plant cystinosin proved too unstable to purify and assay, however, a glycine variant was stable and surprisingly exhibited almost WT transport levels (~90%) (Fig. 4a & Supplementary Fig. 5c). We next analysed the Pro169Gly variant using our binding assay, and discovered that although this variant and the Pro169Ala variant are transport defective, it still retains WT levels of l-cystine binding (Supplementary Fig. 5a). Together, these results indicate the second PQ-motif proline facilitates the conformational changes during transport. The importance of the second PQ-motif proline is perhaps explained through its proximity to Lys166, which sits adjacent to Tyr167 and Asp191 (Supplementary Fig. 7a). Scanning mutagenesis previously identified the equivalent aspartate in the human transporter, Asp305, as being a potential site of proton binding^20^.

To further explore the role of these side chains in cystinosin, we mutated Asp191 to asparagine and Tyr167 to phenylalanine. Interestingly, we observed increased proton flux through the Tyr167Phe variant, as measured using our pyranine-based assay (Supplementary Fig. 7b), indicating that either more protons are moving during the transport cycle or that the kinetics of transport have been altered. However, when we analysed the uptake of radioactive cystine we also observed an increase in the amount of substrate moved (Supplementary Fig. 7c). Further kinetic analysis of the Tyr167Phe variant demonstrated that Vmax increased ~30% relative to WT and the K_M_ increased from 19 μM to 73.3 ± 12.8 μM (Fig. 4b), confirming the role of this side chain in regulating transport kinetics. These results are consistent with the previous analysis of the human transporter and demonstrate that Tyr167 is important for modulating l-cystine recognition^20^. In contrast, the asparagine 191 variant displayed a marked, but slow proton leak and was unable to transport l-cystine, suggesting this variant is uncoupled (Supplementary Fig. 7d). The presence of a slow proton leak indicates a second site of proton binding is involved to gate the transporter, consistent with suggestions from the previous functional analysis using TEVC^20^. Our structure highlighted an interaction between Asp92 on TM3 with Lys221 on TM7 and Gln201 on TM6, which forms the only inter bundle salt bridge between the two 3TM repeats (Fig. 1e). Alanine variants of these side chains proved too unstable to purify (Supplementary Fig. 5c), highlighting their importance for structural stability. To address the role of these side chains in the transport mechanism of cystinosin, we made the equivalent mutations in the human transporter and assayed their function using our TEVC assay (Fig. 4c). Unlike the plant variants, the human proteins all expressed in the oocytes (Supplementary Fig. 6). Our results indicate that whilst the glutamine in the human transporter (Gln315) is not required for function, both the aspartate (Asp205) and lysine (Lys335) are essential and as discussed below, mutation of Asp205 to asparagine in the human transporter leads to infantile cystinosis^41^.

As noted above, a major difference between human and plant cystinosin is the presence of His56 on TM2, which is located close to the disulfide bond of the ligand (Fig. 2b). In the human protein, His56 is a phenylalanine; however, a His56Phe plant cystinosin variant was still functional, displaying ~50% WT activity (Supplementary Fig. 7e). Although this side chain is not essential for transport, the proximity to Lys55 suggested a functional role, which combined with the slightly different pH environments found between the plant vacuole and mammalian lysosome^42,43^, prompted us to study the effects of different pH conditions on the His56Phe variant in the plant transporter. The WT transporter shows transport activity across a wide pH range (6.5–8.2), whereas the His56Phe variant is unable to transport at pH 6.5 but remains functional at pH 7.2 and 8.2 (Supplementary Fig. 7e). As the pH gradient across the plant tonoplast membrane is less steep than observed in mammalian lysosomes, this adaption may have evolved to facilitate more efficient transport under the conditions found within plant cells.

## Discussion

**Fig. 5:**
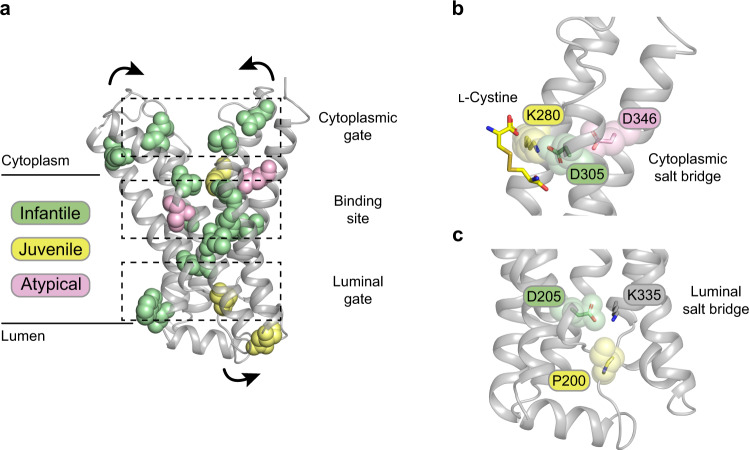
Structural context of cystinosis-causing mutations. **a** A subset of the missense mutations that localise to the cytoplasmic and luminal gates or binding site mapped onto the AlphaFold2 model of human cystinosin. Mutations are coloured according to the severity and onset of disease. Arrows indicate structural movements that occur during transport. **b** Zoomed in view of the cytoplasmic gate with the position of l-cystine superimposed. **c** Zoomed in view of the luminal gate with the inter bundle salt bridge residues highlighted.

The crystal structure of the plant homologue provides a framework for understanding how disease-causing mutations in human cystinosin lead to different severities of cystinosis. Currently, 42 missense mutations have been identified (Supplementary Table 1), which are categorised into four broad groups and relating to age of onset: infantile, juvenile, atypical and ocular^13,15,18^. Omitting those mutations that occur in the N-terminal domain, we can map on the remaining mutations using the model of the human transporter (Fig. 5a and Supplementary Table 1). Many of these mutations map to the functionally important regions, and in particular cluster around the cytoplasmic gate, ligand binding region and luminal gate (Fig. 5b, c).

Previous structures of the bacterial PQ-motif transporters revealed that alternating access transport within this family is likely to occur following the movement of the two 3TM helical bundles around the central ligand, aided by the location of the two PQ-motifs in TM1 and 5^27,28^. However, unlike the bacterial transporters, cystinosin is proton coupled, which introduces the need to couple ligand binding to proton translocation. Our results suggest this occurred in cystinosin through the introduction of two conserved salt bridge interactions that exist on either side of the cystine binding site, which function to couple the open/closed state of the cytoplasmic and luminal gates to proton and cystine binding.

**Fig. 6:**
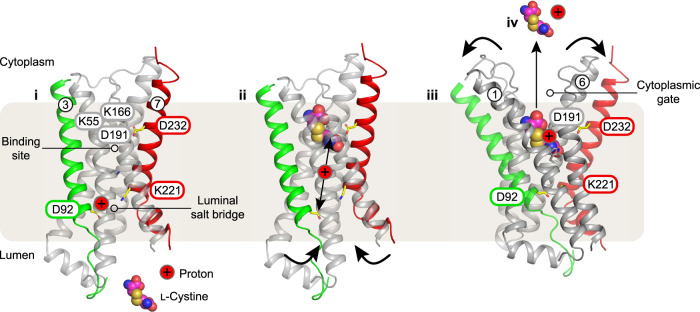
Conserved salt bridge interactions mediate l-cystine transport. Proposed mechanism for proton coupled cystine transport via cystinosin. **i** in the apo state the transporter adopts an outward open state due to protonation of Asp92 on TM3. **ii** cystine binding results in deprotonation of Asp92 and promotes opening of the cytoplasmic gate through interactions within the binding site, with Asp191, Lys55 and Lys166 playing important roles in ligand recognition. Closure of the luminal gate is facilitated through a salt-bridge interaction between Asp92 and Lys221. Movement of Lys221 towards Asp92 would reinforce the movements required to open the cytoplasmic gate as the helices pivot around the bound cystine ligand. **iii** transition to the inward open state occurs and Asp191 become protonated, weakening the interaction with cystine, and promoting release of the ligand and proton into the cytoplasm (**iv**). Deprotonation of Asp191 would create the conditions necessary for the return of the transporter to restart the cycle, facilitated by the proximity of Asp232.

Taken together, our data enables us to propose a working model for proton coupled l-cystine transport (Fig. 6). The transporter adopts an outward open state in the lysosome (state i), with the cytoplasmic gate closed and TMs 1-2 and TMs 5-6 packed together. In this conformation, the luminal gate is open, with TMs 3 and 7 separated. In this state, Asp92 (Asp205; human numbering in parentheses) would be protonated, given the acidic luminal pH. The protonation of Asp92 would function to keep the luminal gate open, as it would weaken the interaction with Lys221 (Lys335). Following l-cystine binding, Asp92 (Asp205) likely deprotonates to engage Lys221 (Lys335), closing the luminal gate (state ii). l-Cystine engages both Lys166 (Lys280) and Asp191 (Asp305), which reside on TM5 and 6 respectively, triggering opening of the cytoplasmic gate, aided through interactions to Trp24 (Trp138) and Lys55 (Lys273). The transporter will transition to the inward (cytoplasmic) open state following the movement of the cytoplasmic ends of TMs 1 and 2 and TMs 5 and 6 away from one another (state iii). There are no salt bridge interactions coordinating the closure of the cytoplasmic gate (i.e., connecting helices of the first and second 3TM bundle), instead the loops connecting these helices contain polar and bulky hydrophobic side chains that pack together to seal the binding site from the cytoplasm. A similar feature is observed in the bacterial PQ-loop homologues^27^. Following opening of the cytoplasmic gate, l-cystine release could be triggered by protonation of Asp191 (Asp305), as this would weaken the salt bridge interaction we observe with the amino terminus of the ligand (Fig. 2c, d). The movement of protons between Asp92 (Asp205) and Asp191 (Asp305) could occur through ordered water molecules, as shown for POT family peptide transporters^44^ or may be facilitated directly by l-cystine. Following l-cystine release, Asp191 (Asp305) would be expected to deprotonate on exposure to the cytoplasm and further protonation of Asp92 (Asp205) would result in the transporter resetting for another cycle. The absence of a pH sensitive element within the cytoplasmic side of the helices suggests this gate is structurally coupled to the luminal gate, which contains the pH responsive element, Asp191. Mechanical coupling between the two gates is achieved by having Lys221 (Lys335) and Asp232 (Asp346) on TM7. Aspartate 232 (Asp346) interacts with Asp191 (Asp305) in the cystinosin structure (Fig. 6), providing a link between movement in the luminal gate to structural changes in the cytoplasmic region of the transporter and l-cystine binding.

Finally, our observation that the first PQ-motif is not required for cystinosin function suggests a division of labour within the transporter, with the second 3TM bundle coordinating a more sensitive interaction network. Certainly, more mutations map to the second 3TM bundle in cystinosis patients, supporting a more prominent role in the transport mechanism (Fig. 5a & Supplementary Table 1). Fusing the two 3TM bundles together clearly represented an important step in the evolution of function within the PQ-loop family and provided an ideal framework to evolve more complicated coupling mechanisms. It would also provide opportunities for oligomerisation and asymmetric assemblies in the membrane, for which we currently have little insight. It will be important to pursue this aspect of PQ-protein biology to fully understand their role in the cell. Nevertheless, the structures and functional data presented, combined with the functional annotation of key disease-causing mutations now provides a firm foundation for developing a molecular blueprint for proton coupled lysosomal cystine transport and cystinosis.

## Methods

### Cloning, expression, and purification of *Arabidopsis thaliana* cystinosin

The gene encoding *Arabidopsis thaliana* cystinosin (Uniprot code P57758) was codon optimised, synthesised (Geneart, ThermoFisher) and cloned into pDDGFP-Leu2d^34^ for expression as a cleavable GFP^His^ fusion in *Saccharomyces cerevisiae* strain BJ5460^45^. Variant forms of plant cystinosin were created by site-directed mutagenesis using overlap PCR. Yeast cells transformed with their respective plasmids were cultivated in synthetic complete medium minus leucine (-leu) with 2% (w/v) d-glucose for 32 h before diluting ninefold into -leu containing 2% (v/v) dl-lactate; wild type protein was expressed using a 15 l fermentation vessel (Eppendorf BioFlo 415) whilst variants were grown in baffled flasks, using 6 l volumes. Following overnight culture, expression was induced at an optical density (OD_600_) of 2.5 by addition of 1.5% (w/v) d-galactose, dissolved in -leu media. After 20–23 h the yeast cells were harvested by centrifugation and lysed using a high pressure (38 kpsi). Following a low-speed centrifuge spin to remove cell debris and unlysed cells (30,000 x *g*) membranes were isolated through two spins at 200,000 x *g* with a resuspension in between using 20 mM HEPES-NaOH, pH 7.5, 1 M K-acetate, and finally resuspended in phosphate-buffered saline (PBS). Wild type cystinosin and variants were solubilized in *n*-dodecyl-β-d-maltoside (DDM, Anatrace) and purified to homogeneity using standard immobilised metal affinity chromatography (IMAC) steps. Solubilisation was carried out for 1 h in 1% (w/v) DDM, 1 × PBS, 150 mM NaCl and 10% (v/v) glycerol. Following ultra-centrifugation at 200,000 x *g* for 1 h at 4 °C, the soluble fraction was supplemented with 25 mM imidazole and bound to Ni-NTA resin (Thermo) in batch for 2.5–3 h at 4 °C. The resin was transferred into a gravity flow column, washed with 25–30 CV of solubilisation buffer supplemented with 0.1% (w/v) DDM and 35 mM imidazole and subsequently eluted with 250 mM imidazole. After overnight dialysis (20 mM Tris-HCl, pH 7.5, 150 mM NaCl, 0.02% (w/v) DDM) at 4 °C in the presence of Tobacco Etch Virus (TEV) protease, a reverse IMAC step was performed using the same resin (1.5 h binding at a final concentration of 10 mM imidazole). The flow-through, containing the purified cystinosin protein, was concentrated using a 50 kDa MWCO centricon (Amicon, Sigma Aldrich) and applied to a Superdex 200 Increase 10/300 GL column (Cytiva) equilibrated in size exclusion buffer (20 mM MES-NaOH pH 6, 150 mM NaCl and 0.03 (w/v) DDM). For crystallisation the detergent concentration was reduced to 0.013% (w/v) DDM. C-terminally Avi-tagged protein was purified as above, and subsequent biotin modification was carried out using the BirA ligase according to the manufacturer’s instructions (Avidity).

### Nanobody selection and purification

To identify cystinosin-specific nanobodies a library was raised through immunisation of a llama using reconstituted protein and screened using C-terminally Avi-tagged and biotinylated protein (method modified from^32^). Specifically, nanobodies were raised in a llama following intramuscular immunisation with purified protein reconstituted into liposomes and using Gerbu LQ#3000 as the adjuvant. Immunisations and handling of the llama were performed under the authority of the project license PPL 70/8108. Blood (150 ml) was collected, and peripheral blood mononuclear cells were prepared using Ficoll-Paque PLUS according to the manufacturers protocol. Total RNA was extracted using TRIzol™ and VHH cDNAs were generated by reverse transcription-PCR using primer as detailed previously^46^. The pool of VHH encoding sequences were amplified by two rounds of nested PCR: firstly with ‘CALL_001’ and ‘CALL_002’, followed by ‘VHH_For’ and ‘VHH_Rev_IgG2’ and ‘VHH_Rev_IgG3’, and cloned into the SfiI sites of the phagemid vector pADL-23c. In this vector, the VHH encoding sequence is preceded by a pelB leader sequence followed by a linker, His_6_- and cMyc-tag (GPGGQHHHHHHGAEQKLISEEDLS). Electro-competent *E. coli* TG1 cells were transformed with the recombinant pADL-23c vector resulting in a VHH library of about 2 × 10^8^ independent transformants. The resulting TG1 library stock was then infected with M13K07 helper phage to obtain a library of VHH-presenting phages. Phages displaying VHHs specific for cystinosin were enriched after two rounds of bio-panning on 50 nM of biotinylated cystinosin, through capturing with Dynabeads™ MyOne™ Straptavidin T1 (Thermo Fisher). Enrichment after each round of panning was determined by plating the cell culture with 10-fold serial dilutions. After the second round of panning, 192 individual phagemid clones were picked, VHH displaying phages were recovered by infection with M13K07 helper phage and tested for binding to cystinosin by ELISA. ELISA positive clones were sequenced and unique nanobodies identified and grouped according to sequence identity. These were analysed by small scale pull-down using the His_6_-tagged constructs as well as co-elution using a Sepax SRT-C SEC-300 size exclusion column (Chromex). The better binders were further characterised using biolayer interferometry using an Octet Red384 (Sartorius) and streptavidin biosensors loaded with biotinylated cystinosin at 20 nM in 20 mM MES-NaOH pH 6, 150 mM NaCl, 0.03% (w/v) DDM to compare k_on_ and k_off_. To calculate the K_D_ a serial dilution of the nanobody was made and following a short baseline step the nanobody was allowed to associate for 10 min followed by a dissociation step of 10 min. Data were analysed in the Octet v9.0 software package and fit to a single binding site model in Prism (GraphPad). All raw data was baseline and reference subtracted, in-step corrected, *y*-axis aligned and filtered with a Savitzky–Golay filter.

### Synthetic nanobody (Sybody) selection

Sybody selection was performed against C-terminally Avi-tagged and biotinylated cystinosin, prepared using the same procedure as above for the llama based nanobody screen. The protocols for sybody selection have been described elsewhere^33,47^. A high affinity sybody, Syb39, was identified from the loop library^47^, which formed a stable complex with the transporter and co-eluted down a Sepax SRT-C SEC-300 column (Chromex). High affinity binders were identified using small scale pull-down assays using the His_6_-tagged binders. Biolayer interferometry was performed on an Octet Red 384 (Sartorius) as described above to compare k_on_ and k_off_ and calculate K_D_. The identified sybodies were cloned and expressed as tag-free binders using the protocols previously described^33^.

### Crystallisation

Crystallisation was performed using protein at 30–37.5 mg ml^−1^ final concentration, as determined using absorbance at 280 nm. l-Cystine was added to a final concentration of 0.5 mM and the protein was left on ice for at least 2 h prior to crystallisation using the lipidic cubic phase method^48^. Protein-laden mesophase was obtained by monoolein with protein in a 60:40 (w:w) ratio using a coupled syringe device (Art Robbins, USA). Initial crystals appeared at 20 °C after 1–3 days in 27–28% PEG 500DME, 100 mM MES-NaOH, pH 5.50–5.75, 100–150 mM K-formate and matured after 5–7 days. Wells were opened using a tungsten carbide glasscutter and the crystals were harvested using 50 or 100 μm micromounts (MiTeGen). Crystals were cryo-cooled directly in liquid nitrogen and stored in unipucks.

### Structure determination

X-ray diffraction data were collected at beamlines I24 and I04, Diamond Light Source, UK (Table 1). Indexing and integration were performed with XIA2 using either the DIALS^49–51^ or autoPROC^52^ pipeline, followed by scaling and merging with AIMLESS^53^. Initial phases were obtained by sulfur SAD phasing. Model building into the electron density map was performed in COOT^54^, with structure refinement carried out using BUSTER^55^. The cystine bound crystal structure (PDB: 7ZKW) contained two molecules in the asymmetric unit and exhibited pseudo merohedral twinning in space group C2 with twin law -h-4/2 l,-k,l applied during refinement using Refmac^56^. The twin fraction was 0.204. The figures and interactions detailed in the study are based on chain B, which gave the strongest density in the refined maps. Geometry restraints for l-cystine were calculated using the grade server supplied by Global Phasing Ltd. Model validation was carried out using the Molprobity server^57^. Images were prepared using PyMol^58^.

### Sulfur-SAD phasing

X-ray diffraction images were collected at the Diamond Light Source I23 beamline^59^ on a PILATUS 12 M detector (Dectris). A single dataset of 360° (rotation increment 0.1°, exposure 0.1 s) was collected at a temperature close to 60 K and a wavelength of 2.7552 Å. The dataset was automatically processed with DIALS^60^ in the *P*2_1_2_1_2 space group to a resolution of 3.2 Å. The structure was solved by sulfur-SAD using the increased sulfur anomalous signal at longer wavelengths, Fast_EP^61^, Anode^62^ and Phenix.autobuild^63^ as implemented in the Diamond automatic downstream processing pipelines.

### Proteoliposome reconstitution

Purified wild type and mutated cystinosin were reconstituted into lipid vesicles consisting of a 3:1 (w:w) ratio of POPE:POPG by rapid dilution. To do so, the proteins were first exchanged into *n*-decyl-β-d-maltoside (DM, Glycon) by gel filtration using the same buffer as before but supplemented with 0.3% (w/v) DM, and subsequently mixed with the extruded vesicles at a 40:1 (w:w) lipid:protein ratio. The lipid protein mix was incubated at room temperature for 20 min before diluting it 27-fold into cold reconstitution buffer (50 mM K-phosphate, pH 7.0). Proteoliposomes were harvested by ultra-centrifugation at 200,000 × g for >2 h and dialysed extensively overnight against two batches of reconstitution buffer. Proteoliposomes were recovered and resuspended to a final concentration of 0.5 μg μl^−1^ as analyzed by SDS-PAGE using ImageJ, comparing all bands of reconstituted protein to a known concentration of detergent-solubilized protein. Proteoliposomes were subjected to two rounds of freeze-thaw using liquid nitrogen and stored at −80 °C. For llama immunisation to raise nanobodies, a total of 600 μg of cystinosin was reconstituted at a lipid to protein ratio of 30:1 and proteoliposomes were resuspended to a final concentration of 1 mg ml^−1^.

### Transport assay using pyranine

Proteoliposomes were pelleted by ultra-centrifugation at 108,000 × g and 4 °C for 25 min and subsequently resuspended in INSIDE buffer consisting of 5 mM HEPES-NaOH, 120 mM KCl, 2 mM MgSO_4_ and 2 mM pyranine (trisodium 8-hydroxypyrene-1,3,6-trisulfonate) at pH 7.2. Proteoliposomes were subjected to eight rounds of freeze-thaw before being extruded through a 0.4 μm polycarbonate membrane. The uniquely formed vesicles were then harvested through ultra-centrifugation as before but at 18 °C and external pyranine was removed by applying the sample to a G-25 column (Cytiva) equilibrated in INSIDE buffer without pyranine. Vesicles were collected again and finally resuspended in INSIDE buffer without pyranine. Transport assays were performed at 25 °C in a cuvette equipped with a small magnetic flea using a Cary Eclipse Fluorescence Spectrophotometer (Agilent) set at dual excitation at 460 and 415 nm and emission at 510 nm. At the start of each assay, vesicles containing 5 μg of cystinosin were diluted into 545 μl of OUTSIDE buffer (5 mM HEPES-NaOH, 120 mM NaCl, 2 mM MgSO_4_ at pH 7.2). After 15 seconds substrates were added per 100 μl from a freshly prepared stock in OUTSIDE buffer (200 μM final if not stated otherwise) and transport was initiated with a final concentration of 1 μM valinomycin (v) in OUTSIDE buffer after 30 seconds. The ratio of the resulting fluorescence profiles were calculated and plotted using Prism (GraphPad) or Excel (Microsoft), transport data from triplicate experiments were normalised to one to allow for comparison. For the pH experiments shown in Supplementary Fig. 6d, the buffer components were as stated above except 5 mM MES was used for pH 6.5 and 5 mM HEPES was used for pH 8.2.

### l-Cystine binding assay

DDM-purified wild type and mutated cystinosin were diluted to 10 μM using a binding assay buffer consisting of 20 mM HEPES-NaOH, 150 mM NaCl and 0.03% (w/v) DDM, also containing up to 3.2 mM l-cystine, set to pH 7.2. After a 5 min incubation step at room temperature the solutions were loaded onto a Prometheus NT.48 (NanoTemper) and nanoDSF (differential scanning fluorimetry) experiments were run at 25% intensity using a 0.5 °C min^−1^ increment. We note that the addition of l-cystine did not significantly increase the melting temperature of the protein. Instead, we found that ligand addition led to a reduction in the tryptophan fluorescence emission ratio measured at 350 over 330 nm before the melting temperature was reached. Ten subsequent data points commencing from 25 °C were averaged and subtracted from a control melt without l-cystine. The fluorescence quench observed from each cystinosin variant with 3.2 mM l-cystine present compared to no ligand was finally normalised by dividing it by that of the average of a WT triplicate. All variants were completed in triplicate using a fresh l-cystine stock for each replicate.

### Radioactive transport assay

Proteoliposomes were pelleted and resuspended in INSIDE buffer (10 mM MES-NaOH, 120 mM K-acetate, 2 mM MgSO_4_ at pH 6.2). Four rounds of freeze-thaw were followed by extrusion through a 0.4 μm polycarbonate membrane. To reduce the volume, vesicles were harvested again at 18 °C and resuspended in INSIDE buffer.

Starting each individual assay, vesicles containing 2 μg protein were diluted into 250 μl OUTSIDE buffer (10 mM MES-NaOH, 120 mM NaCl, 2 mM MgSO_4_ at pH 6.2) also containing 48 μM l-cystine, 2 μM radiocarbon-labelled ^14^C-l-cystine (specific activity 0.2 Ci mmol^−1^, Hartmann Analytic) as well as 10 μM valinomycin (Merck). Assays were performed at 20 °C. 45 μl fractions were removed at different timepoints, diluted into 2 ml of OUTSIDE buffer and vesicles were immediately isolated on a 0.22 μm cellulose filter (Merck) using a vacuum manifold. Filters were washed twice with 2 ml OUTSIDE buffer and subsequently transferred into Ultima Gold (PerkinElmer) scintillation liquid. Remaining radioactivity stemming from the inside of the vesicles was measured using a Wallac scintillation counter.

### Electrophysiology

Human cystinosin without its C-terminal lysosomal targeting motif (GYDQLN) was subcloned with and without a C-terminal GFP into the pFAW vector for microscopy and electrophysiology experiments respectively. Variants of the protein were generated by site-directed mutagenesis and verified via sequencing. In vitro transcription of wild type and mutant *CTNS* was carried out using AmpliCap-Max T7 high yield message marker kit (Cellscript). *Xenopus laevis* oocytes were defolliculated by collagenase treatment. They were then injected with 50 nl of nuclease-free water or 75 ng (50 nl of 1.5 μg μl^−1^ RNA) of human cystinosin wild type or mutant mRNA, and were incubated for 2 days at 19 °C in Barth’s solution (54 mM NaCl, 30 mM KCl, 2.4 mM NaHCO_3_, 0.8 mM MgSO_4_(H_2_O)_7_, 0.4 mM CaCl_2_, 0.3 mM Ca(NO_3_)_2_(H_2_O)_4_, 7.5 mM Tris-HCl, adjusted to pH 7.4) with gentamicin (50 μg ml^−1^)^20^. Two-electrode voltage clamp measurements were carried out on a Roboocyte system (Multi Channel Systems) using 3 M KCl back-filled borosilicate glass pipettes with a resistance of 200–1000 k Ω. Oocytes were voltage-clamped to −40 mV for the measurements. For each oocyte, an initial recording was made with ND-96 solution (96 mM NaCl, 2 mM KCl, 2 mM MgCl_2_, 1.8 mM CaCl_2_, 5 mM MES-NaOH, adjusted to pH 5) without cystine, followed by a second recording in ND-96 with 1 mM cystine (pH 5). For the dose-response curves, these measurement pairs were repeated on a single oocyte for each of the tested concentrations (0–1000 μM). Traces were analysed using the Roboocyte software (Multi Channel Systems). Cystine-evoked current values were obtained by subtracting the mean current after the addition of cystine from the mean current before the addition of cystine. Currents were normalised based on the cystine-evoked currents for the wild type human cystinosin injected oocytes of the corresponding oocyte batch. The data obtained for dose-response curves were normalised based on the maximum response measured (i.e., current at 400 μM or 1000 μM) for that particular oocyte and was fit using Prism (GraphPad). All experiments were repeated on at least three different oocyte batches.

### Fluorescence Microscopy

Oocytes injected with either water, wild type or mutant human cystinosin-GFP mRNA were washed in 1× PBS, stained with 0.05 mg ml^−1^ of CF^®^633 wheat germ agglutinin (Biotium) in 1 × PBS for 5 min at RT, and washed with 1 × PBS. Surface expression was imaged using an LSM-780 confocal microscope (10 × lens) with an Argon (488 nm) and He-Ne laser (633 nm).

### Reporting summary

Further information on research design is available in the Nature Research Reporting Summary linked to this article.

## Supplementary information

Supplementary Information

Reporting Summary

## Data Availability

The data that support this study are available from the corresponding authors upon reasonable request. The atomic coordinates have been in the RCSB Protein Data Bank (PDB) under accession code 7ZK1 (no substrate, Syb39 & Nb4), 7ZKZ (no substrate, Nb1 & Nb4), and 7ZKW (cystine bound, Syb39). Source data underlying Figs. 1b, c, 2e, f, 3b, 4a–c, and Supplementary Figs. 3b, 5a, b, 7c, e is provided as a Source Data file. Source data are provided with this paper.

## Source data

Source Data
