# Supplementary material for: Structural basis for proton coupled cystine transport by cystinosin
Source: Nat Commun. 2022 Aug 17;13:4845. doi: 10.1038/s41467-022-32589-2 (PMC9385667; doi:10.1038/s41467-022-32589-2)
Supplement: Supplementary file 1 — Supplementary Information [file 41467_2022_32589_MOESM1_ESM.pdf]

Supplementary Figures 1-7 & Supplementary Table 1

Structural basis for proton coupled cystine transport by cystinosin.

Mark Löbel[1], Sacha P Salphati[1], Kamel El Omari[2], Armin Wagner[2], Stephen Tucker[3,4], Joanne L Parker[1], Simon Newstead[1,4,]

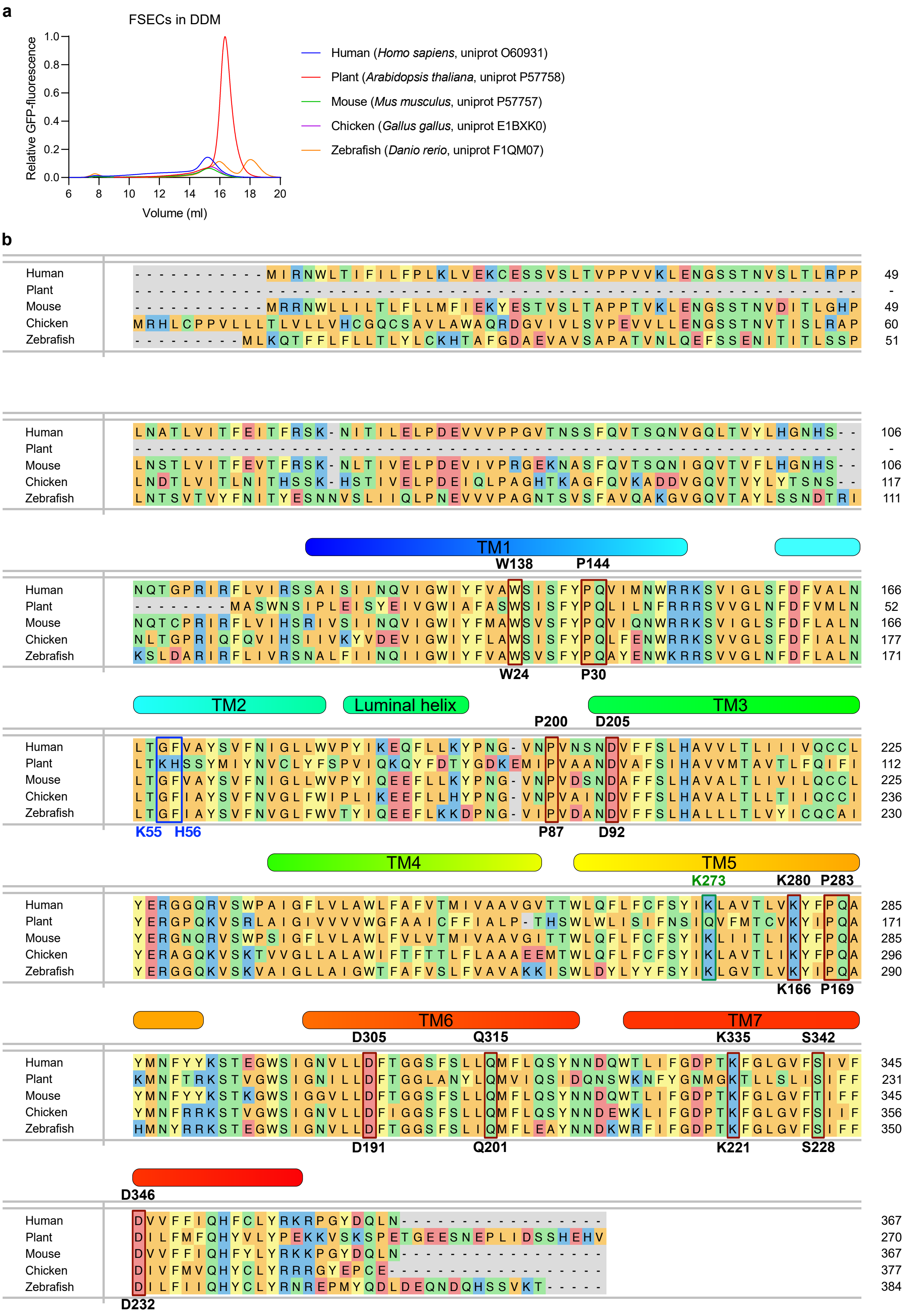

Supplementary Fig. 1: Analysis of cystinosin homologues. a Fluorescence size exclusion chromatography of five cystinosin homologues. Latin names are indicated along with the Uniprot code for each homologue. **b** Sequence alignment of cystinosin homologues (from a) coloured via amino acid chemistry. Residues with functional importance described in the manuscript are highlighted with the human numbering above the alignment and the plant (*Arabidopsis thaliana*) below. Highlighted in blue and green are the major differences between human and plant homologues found within the binding site of the protein.

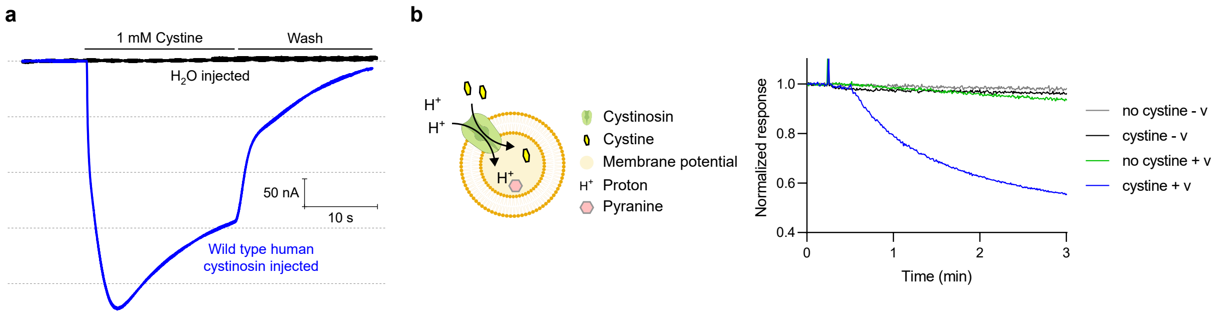

Supplementary Fig. 2: Exemplar data and controls for the assays used in the study. a A typical reading from TEVC on *Xenopus laevis* oocytes used to test human wild type cystinosin and mutant variants. Blue is the trace from oocytes injected with mRNA from WT cystinosin and black is an oocyte injected with water. **b** *Arabidopsis thaliana* cystinosin is a proton coupled cystine transporter. A reconstituted assay system was used to test plant cystinosin and mutant variants reading the change in fluorescence of an intra-liposomal fluorescent dye (pyranine) upon protonation. Cystine was added at 15 seconds and transport was initiated through the formation of a membrane potential through potassium diffusion gradient via the addition of valinomycin (v) at 30 seconds. While there is a very slight proton leak in the system in the presence of just a membrane potential (green - no cystine + v) robust protonation and hence transport is observed with the addition of cystine (blue).

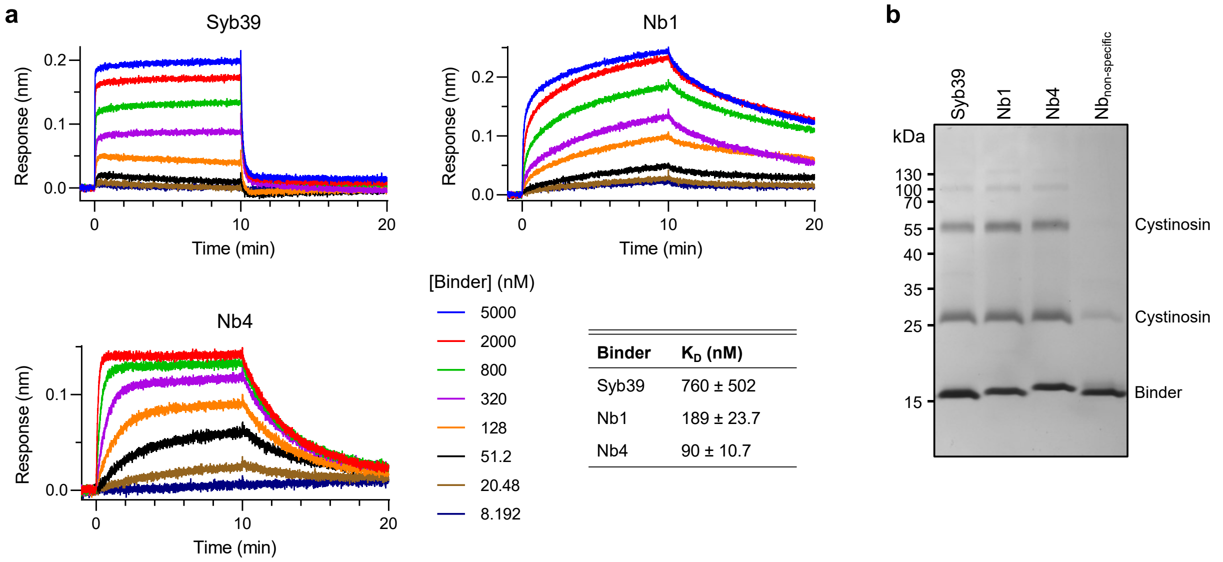

Binder	K_D (nM)
Syb39	760 ± 502
Nb1	189 ± 23.7
Nb4	90 ± 10.7

Supplementary Fig. 3: Characterisation of the binders used in the study. a Representative curves for binding of cystinosin to the binders used in the study as determined using bilayer interferometry. The K_D for each binder was calculated from the mean of three independent experiments with the standard deviation shown. **b** Small-scale nickel-affinity pulldowns using His-tagged versions of the binders were used to screen for interactions between potential binders and cystinosin. Four independent repeats with similar results. Source data are available as a Source Data file.

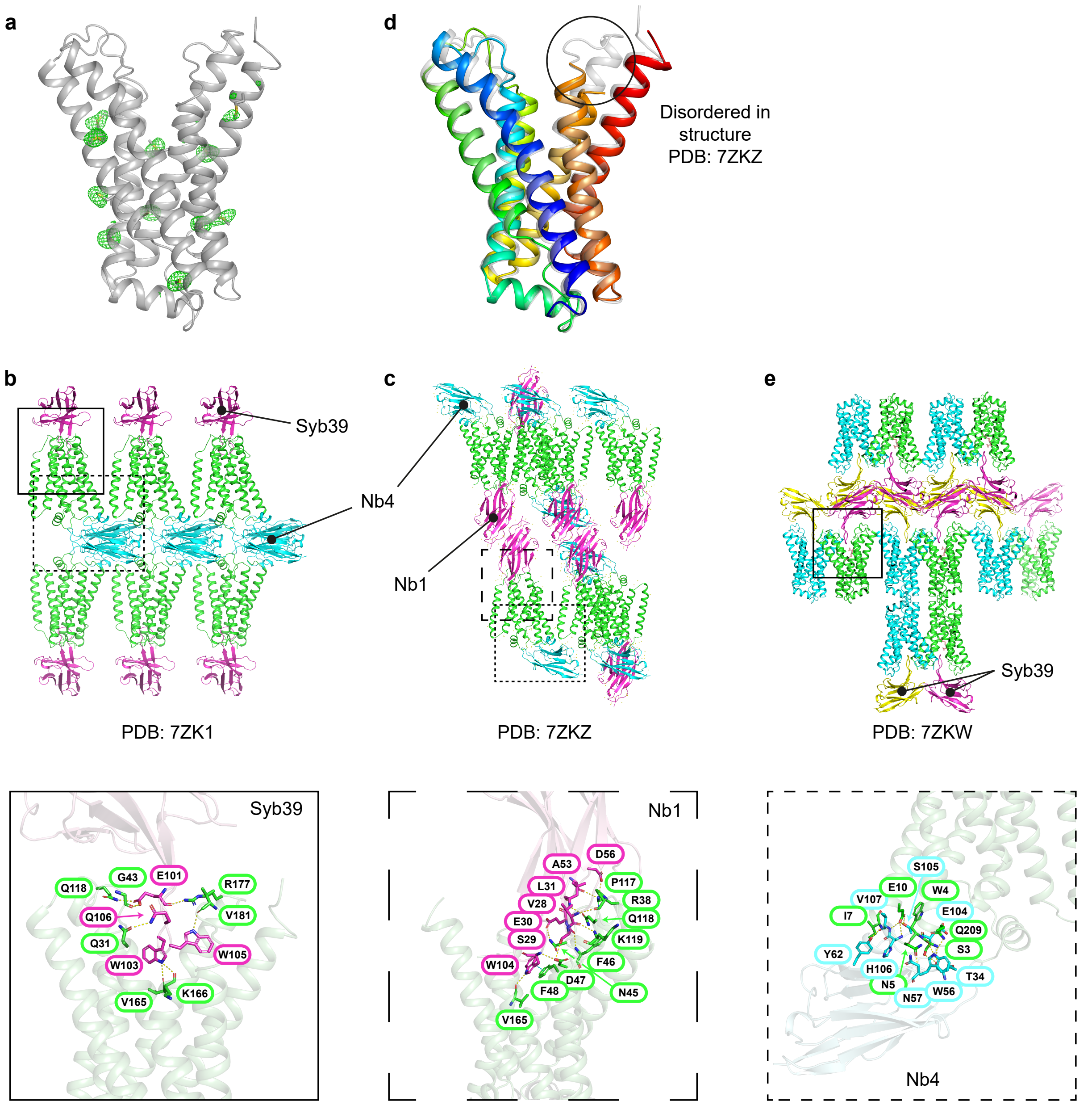

Supplementary Fig. 4: Anomalous difference maps, crystal packing and interactions for binder-cystinosin complexes. **a** Anomalous difference peaks calculated using Anode from data collected on I23 at Diamond Light Source (Table 1). Peaks corresponding to the location of the sulphur atoms are shown (green mesh) contoured at 3 σ mapped onto the final cystinosin model. **b** Crystal packing of structure PDB: 7KZ1, showing the contribution of the sybody and nanobody to the crystal contacts. **c** Crystal packing of structure PDB: 7ZKZ. **d** Overlay of the structures from PDB: 7ZK1 (grey) and 7ZKZ (rainbow) highlighting the region of disorder in the structure obtained from the two nanobody complex. **e** Crystal packing of structure PDB: 7ZKW. Interactions formed between cystinosin and Syb39, Nb1 and Nb4, respectively, are highlighted using yellow dashed lines, π-π stacking is indicated by cyan dashed lines.

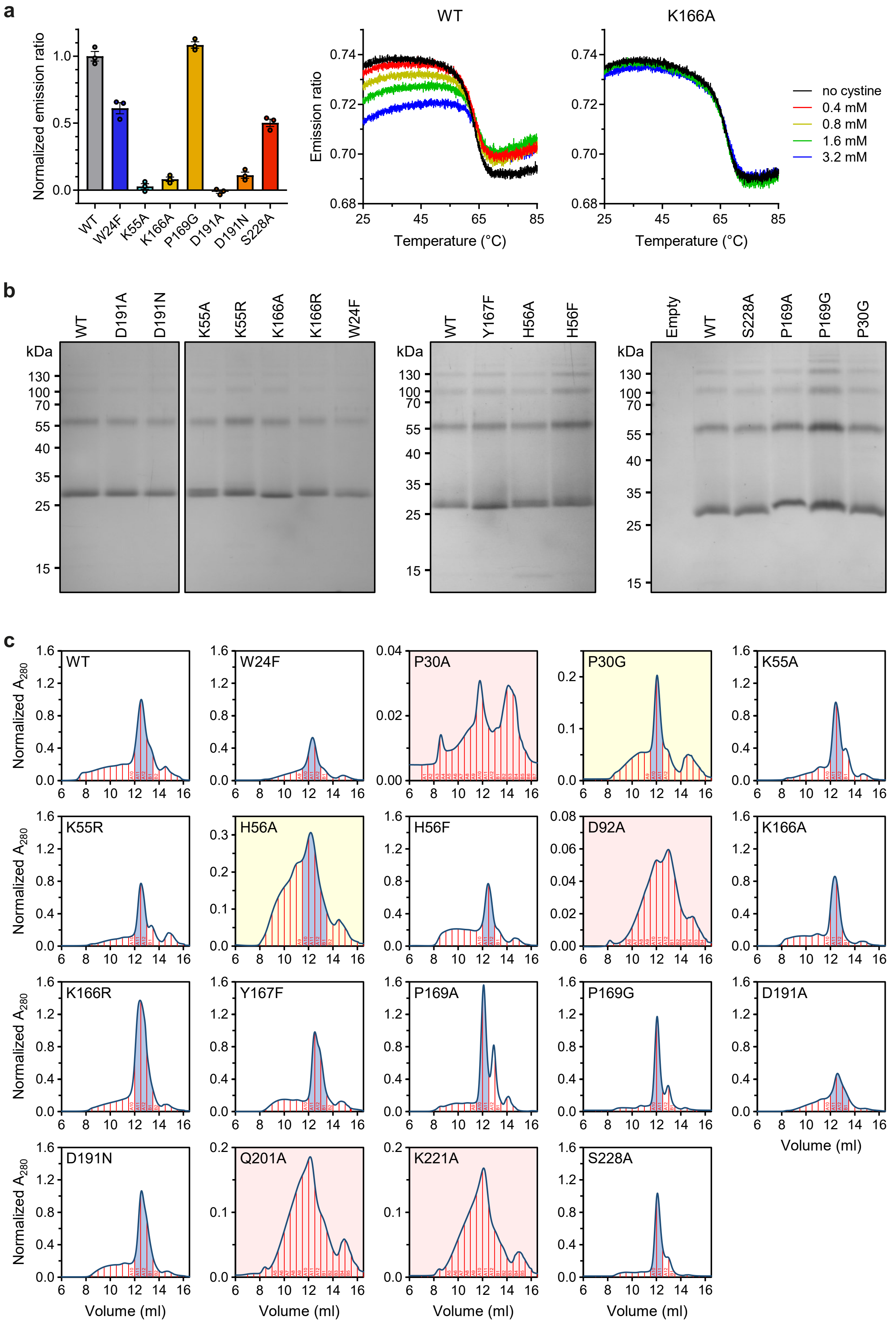

Supplementary Fig. 5: Analysis of the mutant variants. **a** Impact of cystinosin variants on cystine binding using a tryptophan fluorescence quench assay. n = 3 independent experiments for WT and variants, error bars SEM. Typical nano differential scanning fluorimetry (nanoDSF) traces are shown for wild type cystinosin and mutant Lys166. **b** Analysis of WT and variants of plant cystinosin studied in liposome

assays. SDS-PAGE gel of 2.5 μg of re-solubilised protein (in 1% DDM), taken after a 30-minute ultra-centrifugation step at 200,000*g*. Two independent repeats with similar results. **c** Size exclusion profiles of all wild type and mutant variants of cystinosin used in the study. The amount of protein obtained for some of the variants was considerably lower than WT in particular variants Pro30Ala, Asp92Ala, Gln201Ala, Lys221Ala (all coloured red) showed both a low amount of protein and poor quality as judged by the comparatively broad SEC profile. Mutants P30G and H56A (both coloured yellow) expressed lower than WT but yielded enough material for reconstitution. SEC fractions are labelled and pooled fractions are shaded blue-grey. Source data are available as a Source Data file.

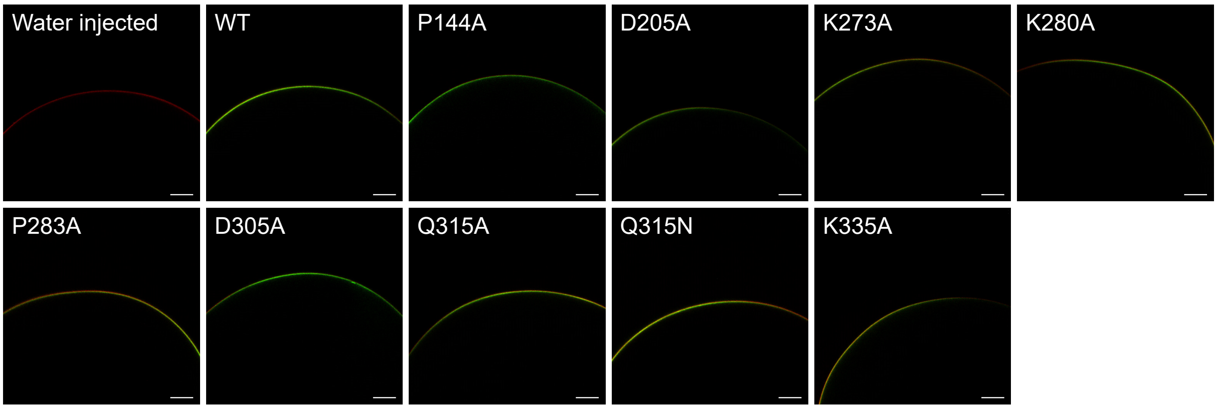

Supplementary Fig. 6: Human cystinosin surface expression in oocytes. Cell surface expression of the human WT and mutant variants of cystinosin in the *Xenopus laevis* oocytes used for TEVC. A C-terminally GFP tagged construct was used for all variants tested and they all localised to the cell surface albeit to slightly different extents; with Pro283Ala, Gln315Ala and Lys335Ala showing lower expression compared to wild type. Scale bar 100 μm. Three independent repeats with similar results.

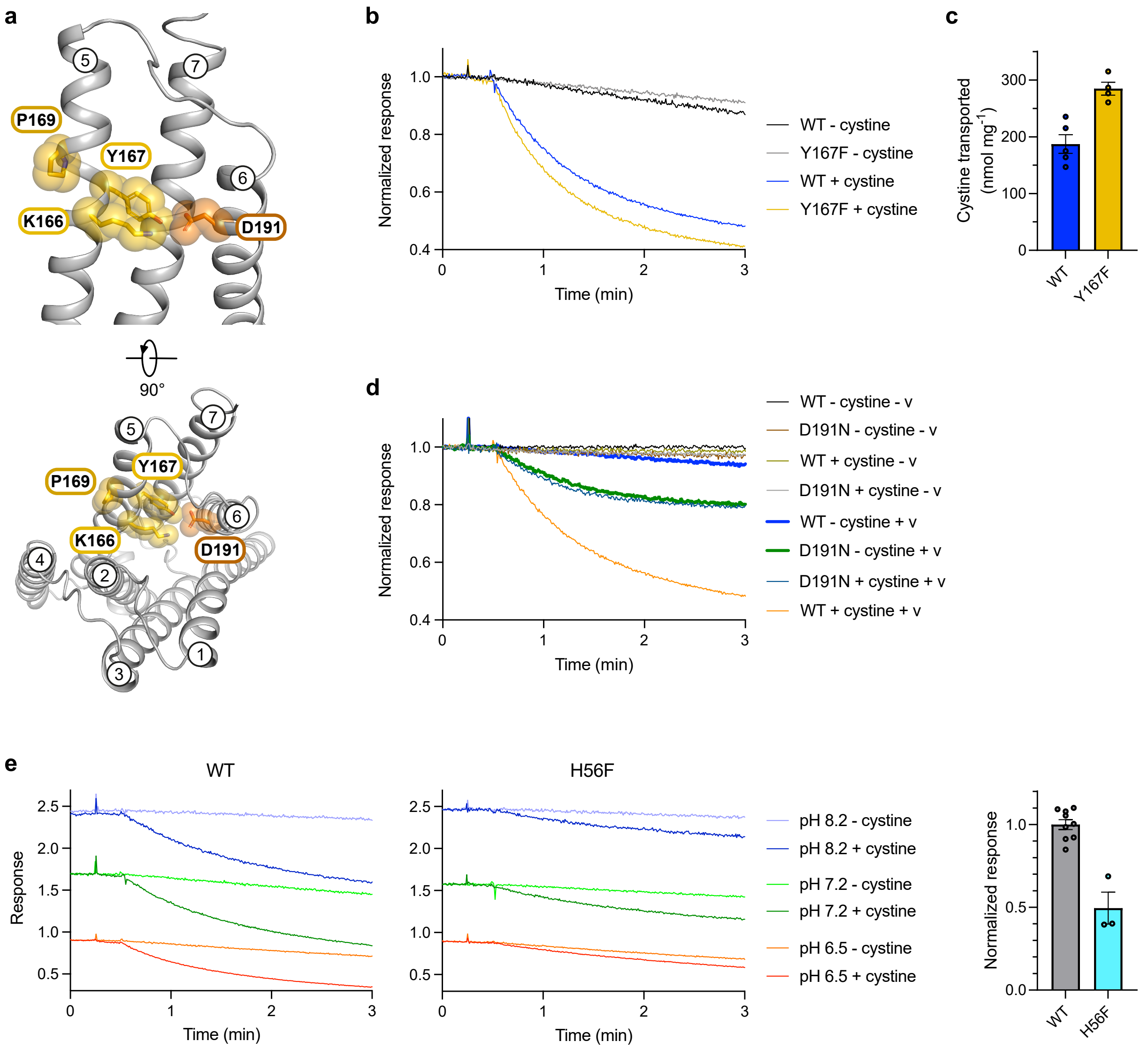

Supplementary Fig. 7: Analysis of Tyr167Phe, Asp191Asn and His56Phe variants of cystinosin. **a** Side and top view of the plant cystinosin crystal structure highlighting the position of Lys166, Tyr167, Pro169 and Asp191 side chains on transmembrane helices 5 and 6. **b** Tyr167Phe variant of cystinosin moves more protons in comparison to WT as determined using a pyranine based fluorescent *in vitro* assay. **c** In addition to the higher transport of protons shown in b, Tyr167Phe also transports more cystine per mg of protein. n = 5 independent experiments for WT and n = 4 for Y167F, error bars SEM. **d** Asp191Asn variant of cystinosin has a marked larger proton leak in the presence of a membrane potential (green line) in comparison to WT (blue line). In addition, this variant no longer responds to the presence of cystine with no additional fluorescence changed on addition of cystine (dark blue line in comparison to the WT, orange line). **e** WT cystinosin (left) functions over a wide pH range in particularly showing robust activity at pH 6.5. Whereas the His56Phe variant (right) shows a very low level of activity at pH 6.5. Representative traces from the pyranine based transport assay at pH 6.5, 7.2 and 8.2 in the presence (200 μM) or absence of cystine. Overall the activity of His56Phe is also reduced compared to WT (pH 7.2 shown). n = 9 independent experiments for WT and n = 3 for H56F, error bars SEM. Source data are available as a Source Data file.

Supplementary Table 1: List of cystinosis-causing mutations.

Mutation	Location	Clinical phenotype
V42I	N-terminal domain	Atypical/juvenile
G110V	N-terminal domain	Atypical
I133F	TM1	Infantile
S139F	TM1	Atypical/juvenile
S141F	TM1	Infantile
R151G	TM1	Infantile
G157D	TM1-2 loop	Infantile
L158P	TM1-2 loop	Infantile
G169D	TM2	Infantile
Y173H/C	TM2	Infantile
N177S/T	TM2	Infantile/juvenile
W182R	TM2	Infantile
K187R	Luminal helix (TM2-3 loop)	Infantile
G197R	TM2-3 loop	Ocular
P200L	TM2-3 loop	Juvenile
D205N	TM3	Infantile
T216R	TM3	Infantile
Q222R	TM3	Infantile
S270F/Y	TM5	Infantile
K280R	TM5	Juvenile
M287I	TM5	Infantile
N288K	TM5	Infantile
S293I	TM5-6 loop	Infantile
S298N	TM5-6 loop	Infantile
D305G/Y	TM6	Infantile
G308R/E/V	TM6	Infantile
G309D/V	TM6	Infantile
N323K	TM6	Juvenile
T334N	TM7	Juvenile
G337R	TM7	Infantile
L338R/P	TM7	Infantile
G339R	TM7	Infantile
D346N	TM7	Atypical/juvenile
G362R	C-terminal tail	Infantile
